# Ethical issues surrounding controlled human infection challenge studies in endemic low‐and middle‐income countries

**DOI:** 10.1111/bioe.12802

**Published:** 2020-08-30

**Authors:** Euzebiusz Jamrozik, Michael J. Selgelid

**Affiliations:** ^1^ Monash Bioethics Centre Monash University Melbourne Victoria Australia; ^2^ University of Melbourne, Department of Medicine Royal Melbourne Hospital Melbourne Victoria Australia

**Keywords:** controlled human infection, endemic, ethics, global health, human challenge studies, research ethics

## Abstract

Controlled human infection challenge studies (CHIs) involve intentionally exposing research participants to, and/or thereby infecting them with, micro‐organisms. There have been increased calls for more CHIs to be conducted in low‐ and middle‐income countries (LMICs) where many relevant diseases are endemic. This article is based on a research project that identified and analyzed ethical and regulatory issues related to endemic LMIC CHIs via (a) a review of relevant literature and (b) qualitative interviews involving 45 scientists and ethicists with relevant expertise. In this article we argue that though there is an especially strong case for conducting CHIs in endemic (LMIC) settings, certain ethical issues related to the design and conduct of such studies (in such settings) nonetheless warrant particularly careful attention. We focus on ethical implications of endemic LMIC CHIs regarding (a) potential direct benefits for participants, (b) risks to participants, (c) third‐party risks, (d) informed consent, (e) payment of participants, and (f) community engagement. We conclude that there is a strong ethical rationale to conduct (well‐designed) CHIs in endemic LMICs, that certain ethical issues warrant particularly careful consideration, and that ethical analyses of endemic LMIC CHIs can inform current debates in research ethics more broadly.

## 1 INTRODUCTION

Controlled human infection challenge studies (CHIs) involve intentionally exposing research participants to, and/or thereby infecting them with, pathogens (or other micro‐organisms) with the primary aim(s) of (a) testing (novel) vaccines and/or therapeutics, (b) generating knowledge regarding the natural history of infectious diseases (and/or asymptomatic infection), and/or (c) developing “models of infection” (i.e., reliable methods [to be used in studies with aims (a) or (b)] of infecting participants with a particular micro‐organism). Such studies are said to be “controlled” because they involve *controlling* the selection and/or production of the micro‐organism strain and the timing, route, and/or dose of infection; infection in a *controlled* environment; infection with micro‐organisms causing no disease or disease that is self‐limiting and/or can be (and is) *controlled* with early diagnosis and/or effective cures/treatments; and/or *controlling* who is being infected (and/or subjected to other experimental interventions).

CHIs can accelerate the development of new vaccines (and therapeutics) because they can be substantially smaller, shorter, and less expensive than other kinds of studies.^1^ Among other advantages, far fewer people need to be given experimental vaccines (that might not turn out to be safe or effective) in CHIs in comparison with vaccine *field trials*, which require many more participants (e.g., up to tens of thousands per field trial as opposed to the <100 participants in most CHIs). CHIs can also provide unique insights into host‐pathogen interactions.^2^

Although several prominent historical cases of unethical research involved the intentional infection of research participants,^3^ CHIs have in recent decades been conducted with research ethics oversight and careful research practices, collectively enrolling tens of thousands of consenting healthy volunteers and involving a wide range of pathogens and other micro‐organisms.^4^ Multiple commentators have argued that controlled human infection can in‐principle be an ethically acceptable research practice, so long as basic research ethics criteria are met.^5^

Although the vast majority of the burden of infectious diseases occurs in low‐ and middle‐income countries (LMICs), CHIs have until recently been conducted almost exclusively in high‐income countries (HICs) where many diseases of interest (e.g., malaria, typhoid, schistosomiasis, etc.) rarely occur. Of the more than 40,000 people that have participated in CHIs in the ~70 years since World War II,^6^ only around 400 participants took part in LMIC CHIs (in just 13 LMIC studies up to 2018)—i.e., less than 1% of the global total.^7^

Potential reasons for the vast majority of CHIs having been conducted in HICs (even for pathogens that are not locally endemic) include: (a) the presence of more/better funded research infrastructure and researchers in HICs;^8^ (b) the greater availability of healthcare resources required to care for CHI participants in HICs, thus providing greater assurance of risk minimization;^9^ (c) the reluctance to conduct research on apparently “vulnerable” populations in LMICs.

The relative current research capacities of HICs and LMICs, including capacity for CHIs, are arguably the result of longstanding injustices in the global distribution of wealth and thus, inter alia, funding for research. This has in turn contributed to a relative neglect of research regarding pathogens that are mainly endemic in LMICs and the perpetuation of large inequities in the global burden of disease. Furthermore, research on HIC volunteers may not always be generalizable to populations in LMICs (e.g., due to population differences regarding naturally acquired immunity, co‐infections, genetics, microbiome, nutrition, etc.), among whom the burden of the relevant disease is often greatest.^10^ There have thus been calls for more CHIs in LMICs in order to remedy such neglect, generate results that are more relevant to at‐risk populations, and build local research capacity.^11^

Despite the potential scientific advantages and other benefits of (LMIC) CHIs, such studies are nonetheless ethically sensitive. Standard research ethics considerations are especially salient in the context of CHIs in endemic LMICs—and analyses of such studies can inform current debates in research ethics (e.g., regarding the acceptability of third‐party risks, appropriate informed consent practices, and appropriate payment of participants) more broadly. This article draws on the findings of a research project that examined ethical and regulatory issues related to CHIs in endemic LMICs via a review of relevant literature and qualitative interviews involving 45 scientists and ethicists with relevant expertise. In what follows we (a) argue that there is a particularly strong case for conducting more (appropriately designed) CHIs in endemic LMIC settings and (b) examine the specific implications of several research ethics issues in such contexts. Throughout, we highlight ways in which the design and conduct of LMIC CHIs can make such studies more (or less) ethically acceptable.

## 2 STUDY DESIGN AND METHODS

### 2.1 Literature review

The details of our literature review are included in the full Final Report of our research project.^12^ Briefly, the review of academic literature and regulatory documents was particularly focused on identifying (a) primary scientific papers detailing LMIC CHIs, (b) relevant historical examples of (other) CHIs, (c) regulatory documents or policy consultations specific to CHIs (whether HIC or LMIC), and (d) bioethical analyses of CHIs and/or ethical issues relevant to CHIs in LMICs.

### 2.2 Qualitative interviews

We conducted qualitative interviews with 45 participants (Table 1). Participants were initially recruited based on (a) involvement in the conduct of CHIs in LMICs, (b) scientific or ethical expertise specifically related to CHIs, (c) expertise in research ethics, and/or (d) involvement in the regulation and/or funding of CHIs research. Further informants were recruited by “snowball” sampling based on suggestions from initial participants. As detailed in Table 1, we recruited a diverse group of informants with different kinds of expertise and based in different locations (in most cases, scientist participants based in HICs had been directly involved in LMIC CHIs).

**TABLE 1:** Characteristics of qualitative interview participants

	*n*	%
**Primary area of expertise**		
Science	33	73.3
Ethics	7	15.6
Regulatory representative	4	8.9
Funder representative	1	2.2
**Primary location of work**		
HIC	26	57.8
LMIC	19	42.2
Africa	6	13.3
Asia	9	20.0
North America	15	33.3
South America	4	8.9
UK/Europe	11	24.4
**Sex**		
Female	20	44.4
Male	25	55.6
**Total**	45	100

As part of the informed consent processes, interview participants agreed to be quoted anonymously (by pseudonym) and/or to waive the right to anonymity and be quoted by name. Interview recordings were transcribed and all data were stored in a secure manner. Transcripts were de‐identified, organized and cleaned before being coded with a combination of pre‐set and open coding. The research team agreed upon an initial code list based on the main aims of the study; and coding then progressed openly and iteratively as emergent codes arose and coding categories were further refined as agreed by the research team. Coded data were analyzed to identify overarching themes and sub‐themes (that were validated through initial member checking in subsequent interviews and via the mechanisms discussed below) with validated themes being used to inform the structure of the Final Report.^13^

### 2.3 Synthesis and validity checking

The findings of the literature review and thematic analyses of qualitative data were synthesized in the Final Report. This paper presents a subset of themes highly relevant to the ethical acceptability of CHIs in endemic LMIC settings (see Table 2) and includes a selection of interview data. Draft copies of the Final Report were shared with (a) a subset of participants who provided feedback to the research team (enabling an assessment of internal validity) and (b) participants at two international meetings of researchers and policymakers with relevant expertise (enabling an assessment of external validity and transferability).^14^ Comments were incorporated and/or addressed as appropriate (in most cases with de‐identified acknowledgement in light of participants’ wishes).

**TABLE 2:** Themes and subthemes in the qualitative interview data set of the larger project; themes covered in this paper in italics

Theme	Subthemes: challenge studies in general	Subthemes: endemic settings/LMICs
**Scientific justification**	‐ *Accelerate* */improve vaccine development* *Selecting vaccine candidates* ‐ *Identify correlates of protection* ‐ *Develop models of infection* ‐ *Study pathogenesis, immunity, and transmission*	‐ *Justification in endemic settings/LMICs* *Improved generalizability* *Unique results* *Capacity building* *Responsiveness to local health problems*
**Public health benefit**	‐ *Acceleration of vaccine development* ‐ *Studying pathogenesis, immunity, and transmission* ‐ Maximizing data collected per challenge	‐ *Improved generalizability (developing vaccines for target populations)* ‐ *Capacity building*
**Participant risk**	‐ Risks related to challenge infection ‐ Burdens related to participation Mental health risks ‐ Risks related to absconding ‐ Limits to risk Long‐term risks, lasting harms, and rare but severe harms Comparison to organ donation Analogy to higher risk employment Comparison to Phase I drug trials	‐ Reduced risk in endemic settings ‐ *Comparison to background risk of infection* ‐ Ability to access healthcare outside the study
**Direct benefit to participants**	‐ Lack of direct participant immunity benefit in non‐endemic settings	‐ *Immunity as direct benefit*
**Third‐party risk**	‐ Right to withdraw ‐ Co‐ordination with local public health agencies	‐ *Need for adequate research and/or public health infrastructure* ‐ *Third‐party risks related to insect vectors of vector‐borne diseases* ‐ *Comparison to background risk of infection* ‐ *Site selection: endemic vs. non‐endemic areas within LMICs* ‐ Comparison between challenge strain and locally prevalent strains
**Participant selection**	‐ Implications of selection criteria for generalizability of results ‐ Altruism among participants ‐ *Recruitment of students* ‐ Recruitment of (other) vulnerable populations ‐ Need for more data on participant motivations	‐ *Altruism among LMIC participants* ‐ *Improved generalizability in endemic settings* ‐ *Selection related to immunity and/or innate resistance* ‐ Recruitment of HIV positive individuals
**Children**	‐ Need for prior safety data from adults ‐ Generalizability from adults to children ‐ Reputational risk ‐ Inducement of parents	‐ Potential scientific rationale for infections primarily affecting children ‐ Consideration of local views regarding research with children
**Payment of participants**	‐ Rationales for payment ‐ Models of payment ‐ Potential for undue inducement ‐ Recruitment of underprivileged groups ‐ *Over‐volunteering* ‐ Comparison to other types of work/labor ‐ Appropriate levels of payment	‐ *Locally appropriate levels of payment* ‐ *Inducement in the context of severe poverty* ‐ *Cultural views regarding payment* ‐ *Local sustainability of research payment*
**Expert review**	‐ Benefits and limitations of: Standard institutional review Multi‐center review National review Independent expert review Need for expertise related to: Relevant pathogen(s) Challenge studies Research ethics ‐Risk‐benefit assessment ‐Conflicts of interest	‐ Importance of local review and approval ‐ Capacity building of ethics review expertise
**Consent**	‐ *Education level and consent* ‐ Vulnerability and consent ‐ Undue inducement Accepting risks Concealing health information ‐ Test of understanding ‐ Understanding of third‐party risks	‐ Translation of consent into local languages *‐ Greater knowledge of disease in endemic settings* ‐ Avoiding labeling entire LMIC populations as “vulnerable” ‐ Information‐giving during community engagement (prior to consent)
**Community engagement**	‐ Conditions under which engagement particularly useful or necessary ‐ Definition of relevant community ‐ Need for mutually informative engagement between researchers and community	*‐ Appropriate community engagement for particular settings* *‐ Collaboration between clinical and social scientists* *‐ Understanding local attitudes to research, vaccines, payment of participants* *‐ Addressing local concerns and rumors*
**Regulatory considerations**	‐ Regulation of: Research Challenge strains Experimental interventions (vaccines, treatments) ‐ Lack of international standardized regulations of challenge strains	‐ Lack of specific local regulation of challenge strains ‐ Capacity building of regulators

## 3 ETHICAL ISSUES RELATED TO ENDEMIC LOW‐ AND MIDDLE‐INCOME COUNTRY CONTROLLED HUMAN INFECTION STUDIES

Controlled human infection studies raise a number of ethical issues, many of which are familiar within research ethics discourse more generally (and/or discussed elsewhere in this Special Issue), though they may have specific implications in the context of (LMIC) CHIs (see Table 2). Assuming CHIs are, at least in‐principle, ethically acceptable an initial question relates to the degree of ethical justification for conducting a particular (type of) study in a particular (type of) setting. Below, we consider why there may be an especially strong case for conducting (appropriately designed) CHIs in endemic settings, presenting several reasons raised by interviewed experts. We show that certain ethical issues related to their design and conduct nonetheless warrant particularly careful attention in later sections of this paper that focus on (a) potential direct benefits for participants, (b) risks to participants, (c) third‐party risks, (d) consent, (e) payment of participants, and (f) community engagement. Throughout, we highlight (a) ways in which the design of LMIC CHIs can make such studies more (or less) ethically acceptable and (b) the implications of analyses of LMIC CHIs for current debates in research ethics.

### 3.1 The case for controlled human infection studies in endemic settings

Given that CHIs are often expected to lead to significant public health benefits more efficiently (e.g., in terms of time, costs, and number of research participants) than alternative research designs, some commentators have argued that, beyond such studies being merely ethically acceptable, there is an ethical imperative to conduct CHIs if/when no other (less burdensome) feasible research design could obtain equally valuable results and if/when *not* performing CHIs could lead to greater net harms including (a) longer delays to the development and implementation of beneficial new interventions for (neglected) infectious diseases and/or (b) the exposure of more participants to potentially greater risks in alternative study designs (e.g., field trials).^15^

If the case for conducting CHIs (in general) is grounded (in part) in the need to relieve significant burdens of (neglected) infectious diseases (as efficiently as possible), then there is arguably an even stronger ethical case to conduct CHIs in endemic LMICs in particular because, inter alia:

1. CHIs in endemic LMICs may be more scientifically valid and/or efficient in terms of producing results that are relevant to disease control in at‐risk populations, largely because the results of CHIs performed in (non‐endemic) HIC populations may not always be generalizable to (endemic) LMIC populations.
2. While CHIs are commonly considered to be non‐therapeutic research involving healthy volunteers that do not have potential to benefit directly from research participation, in at least some cases (as discussed below in 3.2.1) participants in endemic LMIC CHIs might benefit directly from research participation.
3. Endemic‐region CHIs are more likely to recruit individuals drawn from populations that stand to benefit from (any interventions developed as a result of) the research (i.e., the burdens and the benefits of the research occur in the same or similar populations).
4. CHIs may be less risky to some participants in endemic regions (as discussed below in 3.2.2).
5. Building local capacity for (infectious disease) research in LMICs may help to increase the degree to which research addresses neglected diseases (which are predominantly endemic in LMICs).

There are thus strong ethical reasons that support conducting (more) LMIC CHIs. However, even if there is an especially strong case for conducting such studies, certain (other) ethical issues related to their design and conduct warrant particularly careful attention. This is because (a) CHIs may sometimes involve, or at least be perceived to involve, particularly high levels of risks (for participants and third parties) and other burdens for participants (and such studies must therefore be carefully designed and conducted to ensure that expected benefits outweigh risks and burdens); and (b) local and/or international community acceptance of CHIs being conducted in endemic LMICs may be contingent on such studies being designed and conducted to especially high ethical (and scientific) standards; and (c) certain ethical considerations, though familiar in research ethics discourse, may have particular (underexplored) implications in the context of endemic LMIC CHIs. The evaluation of these latter implications may both improve the design and conduct of LMIC CHIs and/or provide novel case studies relevant to ongoing debates in research ethics.

In the next section we address each of the following topics and their specific implications in the context of endemic LMIC CHIs: (a) the potential for direct participant benefit, (b) the potential risks for participants, including the evaluation of such risks in light of background risk, (c) the potential risks to third parties, (d) appropriate informed consent practices, (e) appropriate payment of participants, and (f) appropriate community engagement and its relevance to local community acceptance of CHIs.

### 3.2 Specific implications of ethical considerations

#### 3.2.1 Potential individual benefits for participants

CHI participants in non‐endemic settings would usually have little or no chance of benefiting directly from controlled infection with pathogens (which they would not encounter in daily life). However, if a person is at high risk of infection with the relevant pathogen in daily life (e.g., in endemic settings), being infected in the course of CHIs will often (a) entail less risk than being infected “in the wild” (e.g., because of more immediate diagnosis and comprehensive medical care) and in some cases (b) confer a benefit in terms of immunity (whether partial or complete/”sterile”) similar to that of vaccination (albeit achieved with a comparatively higher risk intervention),^16^ that will reduce the likelihood and/or severity of future bouts of infection.^17^

Such considerations of individual benefit have not been widely discussed in the CHIs ethics literature, perhaps because modern CHIs have usually taken place in HICs with pathogens that are not locally endemic. In a recent exception, the 2017 Report on Ethical Considerations for Zika Virus Challenge Trials does mention possible benefits of this kind for challenge study participants recruited in endemic regions during periods of significant transmission,^18^ and the possibility of such benefits was also noted by Michael Selgelid in a presentation at the 2013 Wellcome Trust Scientific Conference on Controlled Human Infection Studies in the Development of Vaccines and Therapeutics.^19^ CHIs might also lead to direct benefits for individual participants in endemic settings if they involve the testing of a vaccine candidate that turns out to be efficacious. Several stakeholders appealed to such considerations; for example:

[The] rationale for participating in research is that … you may help yourself and you may help your community and you may help the world and if you do it in … a non‐endemic country, then it’s just the last one of those; whereas [for CHIs in endemic settings] it’s probably all three because, maybe, there is a small chance that an individual volunteering here for a [CHIs] may benefit, in terms of enhanced immunity. [Scientist, Asia]

Still, whether or not there are any potential direct benefits, most CHIs to date arguably impose increased risk to participants overall, and it would be unusual if infection as part of a challenge study entailed an expected *net* benefit. As one interview participant noted:

[The degree to which an individual participant can be said to benefit] depends on the attack rate where you are and what the probability is [of being infected in daily life, as compared with participating in CHIs]. [I]f you [participate in a] challenge [study] you’ve got a definite risk of infection and an unknown risk of severe complications. [Scientist, UK/Europe]

Nonetheless, the prospect of individual benefits of participation is at odds with standard views of CHIs as a type of research involving no potential direct benefit for healthy volunteers. In an endemic setting, CHIs might lead to direct benefits for participants and, in such cases, be (arguably) more ethically acceptable than non‐endemic CHIs, other things being equal.

#### 3.2.2 Risks to participants

Conducting CHIs in endemic LMICs might also involve different risks for participants (as compared with HIC CHIs). On the one hand, there might in some cases be greater risks in (outpatient) CHIs where local healthcare and other infrastructure is fragile—for example, one recent malaria CHIs in Nairobi, Kenya, was designed as an inpatient study (whereas many comparable malaria CHIs in HICs are primarily outpatient studies) because there were concerns that heavy traffic would mean that participants who developed malaria symptoms would not be able to receive healthcare in a timely manner.^20^ Such risks can be minimized by ensuring study infrastructure is adequate, and by using inpatient study designs where necessary (as in the Kenyan study). Although this may entail greater costs, it would arguably be worth expending more resources in order to ensure that LMIC CHIs participants are not unduly exposed to greater risks than their HIC counterparts in similar studies.

There might still be concerns that the risk of CHI participation could be higher for some individuals (e.g., those with inadequately treated comorbidities or co‐infections, particularly in LMIC populations with many unmet health needs). However, CHIs (including in LMICs) generally recruit healthy volunteers (in order to reduce risks to participants) and thus these population‐level health differences in LMICs as compared to HICs would not likely result in higher risks for participants if, as would be expected, those with such health issues are excluded.^21^

On the other hand, there will often be cases where endemic‐region CHIs are actually less risky for participants. For example, CHIs could involve lower risks of severe disease (during participation) if they recruit individuals who have (partial) acquired immunity due to prior infection^22^ and/or innate forms of resistance to particular pathogens (e.g., genetic conditions affecting red blood cells such as sickle cell that reduce the severity of malaria^23^).^24^ As one scientist observed:

Those who have been exposed to malaria are not likely … to end up with severe disease [as a result of study participation] because they have been [exposed before and developed immunity] and … [this is] a much safer population to deal with because the risks are much lower. [Scientist, Africa]

##### Background risk and risks to participants

Furthermore, where participants in a challenge study are at risk of being infected with a pathogen in daily life (e.g., because they live in an endemic area^25^), in some cases this background risk reduces the risk an individual would take on by participating in a challenge study.^26^ It may thus be more ethically acceptable, from the point of view of balancing the risks and benefits of a study, to enroll those who already face higher background risk (other things being equal). There was widespread agreement among interviewees that such considerations could be ethically relevant in terms of minimizing risk in CHIs study design and might often favor conducting CHIs in endemic populations. For example, Prof. Jonathan Kimmelman argued that: “[T]here are some compelling reasons [to conduct endemic‐region CHIs], and that’s one of them, that … the background prevalence means there is less of a differential … between the [alternative] of not participating and … deliberate exposure.”

Such considerations were part of the explicit justification for early challenge studies conducted with yellow fever (in endemic settings) in the early 20th century, which are still widely regarded as ethically acceptable despite the high risks of participation.^27^ Background risk was also part of the justification of the Willowbrook hepatitis challenge studies (in which institutionalized children were infected with viral hepatitis, which was endemic to their overcrowded institution), although this program is widely regarded as being ethically *unacceptable* in light of current norms. Criticisms of the Willowbrook studies have in part focused on the degree of background risk, since the lower the degree of background risk, the higher the additional risk individuals would be facing/accepting as a result of study participation (see discussion above regarding there being a net increase in risk of participation despite potential individual benefits).^28^

Research ethics literature regarding background risk more generally^29^ provides reasons for being wary about the sentiment that risk imposition on participants might be more acceptable where background levels of risk are higher if/when (a) higher levels of background risk (e.g., in LMICs) themselves reflect injustices and/or (b) research participation would significantly increase risks to participants who already face high background risks (while, as above, it should be kept in mind that *the absolute magnitude of risk increase is a key consideration*, independent of background risk magnitude). Part of the point of (b) is that those who favor a Rawlsian account of ethics/justice, which requires making the worst off groups of society as well off as possible, might conclude that, other things being equal, it is more acceptable to impose higher research risks on well‐off participants in HICs (with lower background risks) than to impose lower marginal risks on less well‐off participants in LMICs (with higher background risks)—because we should avoid worsening the situation of those who are already worst off. A second point of (b) is that if the net increase of risk resulting from CHIs participation is high enough for those who already face high background risks, then CHIs may not be justified even if the net increase in risk for such participants is lower than would have been the case for participants elsewhere: a comparatively lower level of increased risk increase does not entail an acceptable level of increased risk (if the lower level of risk increase is itself quite high).

Although background risk of many infectious diseases in LMICs arguably do reflect historical injustices,^30^ appropriately controlled human infection studies generally involve low net increase in risk to participants^31^; furthermore, one reason to conduct (more) LMIC CHIs is to remedy historical injustice and neglect of LMIC‐endemic pathogens (and to do so more efficiently). Thus, if background risk is relevant to risk‐benefit assessments regarding CHIs, it would arguably often be (more) ethically acceptable to conduct such studies in endemic settings since conducting CHIs in non‐endemic settings would potentially involve higher net increase in risk to participants (as well as a sometimes lower prospect of generalizable public health benefits).

In summary, if risks are minimized through appropriate study design (e.g., the development/use of appropriate study infrastructure and the exclusion of individuals with relevant comorbidities) CHIs in endemic settings will involve at most the same risk and, in many cases, less increased risk to participants (e.g., for those with prior immunity and/or innate resistance) as compared with those in non‐endemic settings.

#### 3.2.3 Third‐party risks

Depending on how a study is designed (and on local epidemiological factors) CHIs may be associated with some level of risk of transmission of the challenge infection from participants to third parties. Regarding such risks in infectious disease research more generally, some have argued that investigators have significant ethical duties to third parties, extending even to the need to seek consent from (identifiable) potentially at‐risk third parties before commencing a study.^32^ One way to obviate the need for such additional consent procedures is to reduce third‐party risks to near zero by (a) rigorous infection control and biosafety procedures at CHIs research centers, and, in some cases (b) strict isolation of participants (e.g., by keeping them in an “inpatient” setting for the period during which they are potentially contagious), although this in turn may entail significant burdens for participants.

In any case, the potential risks may vary in different contexts depending on the mode of disease transmission. For vector‐borne diseases such as malaria, if there are no local vectors then there are minimal risks of third‐party transmission (apart from blood donation by participants while infected). Thus, several LMIC malaria CHIs have been conducted in cities in endemic countries where there are no malaria vectors (so as to minimize third‐party risk).^33^ Similarly, the risks of transmission of diarrheal pathogens via sewerage systems may be low in HICs with adequate sanitation, but could be higher in communities with poor access to sanitation (e.g., in LMICs), suggesting a strong rationale for inpatient studies and/or robust biosafety procedures in such settings; both approaches have been followed in CHIs involving diarrheal pathogens in Thailand.^34^

##### Background risk and third‐party risk

An endemic setting by definition entails that participants *and third parties* will face a background risk of being infected with the pathogen in question in day‐to‐day life. Thus, the potential for third‐party risks in such contexts can lead to controversial questions. How important, for example, is a small third‐party risk and/or single episode of transmission (e.g., from a study participant to a third party) in the context of high local endemic transmission (and/or high average local levels of immunity)? Some individuals and communities may consider this additional risk negligible, while others may see each additional episode of transmission as highly significant—the views of stakeholders interviewed for this project held widely divergent opinions on this matter. For example, one African scientist noted that third‐party risks in many CHI designs are low (e.g., because of control of the pathogen) and arguably not significant in highly endemic settings:

[If] there is not much greater risk [to third parties, compared to background risk] and you are not using a strain that is resistant to any of the drugs that are available, then people [once they understand this] will be much more comfortable I think … most of the risk[s] that we see are much more academic than real [or] practical. [Scientist, Africa]

In contrast, one HIC researcher emphasized the potential significance of low probability but high severity outcomes and the fact that such third‐party risks can be prevented:

[C]ontainment is possible. It’s expensive. Not so expensive in developing countries as it is in developed countries, but it’s possible and if you can minimize risk [to third parties] you should do so, and remember that it’s a drop in the ocean, but it’s a drop in the ocean that can result in death. [Scientist, UK/Europe]

Given this potential controversy, and given the potential for third‐party risks to undermine public trust in research, the potential for such risks would constitute an additional reason for robust community engagement (to assess community views on the importance of such risks and/or to seek “community consent” for the research to proceed) and for carefully designed research procedures that reduce third‐party risks.

#### 3.2.4 Consent

Recent CHIs in both HICs and LMICs have generally been conducted with relatively comprehensive and stringent consent processes, involving multiple information sessions for participants, and tests of understanding to ensure that prospective participants comprehend important aspects of the study before consenting to enrolment.^35^ Given that such processes involve complex information, it is sometimes thought that it would be more ethical to recruit those with higher levels of education as research participants because this may improve informed consent (e.g., if educated participants more easily understand information about the study). Some CHIs (including in LMICs) have thus aimed to recruit tertiary‐educated individuals and/or university students (especially medical students) in particular.^36^ Despite these apparent advantages, there are also several ethical disadvantages of such a recruitment strategy: (a) excluding less educated individuals might be unjustified if they are able to understand a study well enough to provide adequate informed consent, (b) university students (or those who have received university education) may not be representative of the eventual target population for an intervention (e.g., because they are more likely to be affluent and/or to live in cities and less likely to live in highly endemic parts of LMICs and/or because in some countries women are much less likely than men to receive university education), (c) excluding less well educated individuals from CHIs research may thus be unfair, especially where poor and/or less well educated and/or female individuals are at higher risk of the disease in question (yet excluded from research regarding a given pathogen), (d) students may feel pressure to participate (e.g., from academics within the faculty with an interest in the study) making consent less voluntary, and (e) educated individuals (e.g., healthcare workers) may sometimes actually be less compliant with study protocols than other potential participants.^37^

In practice, LMIC investigators have sometimes been successful in recruiting enough tertiary‐educated individuals for CHIs,^38^ whereas others have found it difficult to recruit as many students as planned (and thus recruited others with lower average education levels).^39^ Social scientists embedded with some recent challenge studies have suggested that many less educated individuals appeared to be able to provide adequate informed consent, especially with well‐designed community engagement and multiple opportunities for careful explanation of the study.^40^ As one researcher noted:

There were all kinds of education levels. [Our work with these participants] helped us to … realize it does not necessarily have to be the level of education that mattered, it’s about understanding what the key elements … of this study are. [Scientist, Africa]

Such observations arguably undermine the presumption in favor of recruiting especially well‐educated individuals. Furthermore, including less educated individuals can help researchers to recruit more people from rural, highly endemic areas, and thus learn more about acquired immunity and the efficacy of interventions in those at particularly high risk of the infection in daily life.^41^ This might have implications for the ethical acceptability (and/or scientific validity) of particular studies in terms of the public health benefits they aim to achieve (as well as in terms of the individual risk and benefit considerations discussed above), as outlined by Dr. Meta Roestenberg, in her interview for this project:

If you’re developing a vaccine, and you’re planning to actually deploy that in super rural areas where the majority of the population is illiterate, obviously you would want to move that controlled human infection model to that population also because that population for lots of scientific reasons might respond differently so you need to … research whether it’s going to work in that population as well.

There may be one additional way in which recruiting those who live in or near highly endemic areas, even if they are less educated, is ethically preferable (so long as adequate informed consent is assured): such individuals may be more likely, on average, to have an interest in the goals of the research because prior experience of the infection in question (e.g., in their own lives or in those of local community members) may lead to greater understanding of the need to reduce the harms of such (familiar) infections and thus motivate participation in research, above and beyond a more general sense of altruism that may motivate individuals in non‐endemic areas.^42^ Prof. Jonathan Kimmelman cited this consideration with regards to Zika virus CHIs in epidemic/endemic settings:

[In] a country like Brazil, where Zika has been a problem … there are people who are willing to … be soldiers against the disease that they see afflicting … their peers, as opposed to … a low income person in Baltimore, who is unlikely to get Zika exposure, unlikely to know someone who has Zika, and is [participating in research] to pay the rent.

In summary, (a) presumptions in favor of recruiting only tertiary‐educated individuals may not always, on balance, be ethically justified; and (b) those in endemic settings who are familiar with the disease in question may be well placed to understand the risks inherent in CHIs and may also be more motivated to participate in research aiming to reduce the (local) burden of relevant diseases.

#### 3.2.5 Payment

Many LMIC populations in endemic areas have relatively high levels of economic disadvantage as compared with those in (non‐endemic) HICs. Thus, issues related to payment and/or undue inducement may be (or be perceived to be) particular concerns in LMICs (although such concerns are not unique to CHIs). With regard to payment, participation in CHIs often involves significant time commitments and other burdens for participants (including, in some cases, long inpatient stays) and, in HICs, attracts relatively high levels of payment. There was widespread agreement in our qualitative data that it is considered appropriate to provide payment to participants, including those in LMICs, in order to offset these burdens. As one HIC scientist suggested:

Often the procedures for challenge studies are really quite onerous compared to other studies so if you just add all that up together, just logically, the amount that they should be paid is more than for other studies. How much that should be should probably be linked to local purchasing parity. That makes sense to me. [Scientist UK/Europe]

Many interviewees had observed a status quo that (higher levels of) payments for study participation were widely accepted for HIC participants but were considered unacceptable in LMICs, reflecting an arguably unjustified double standard. As one African CHIs researcher noted:

[F]or a long time, in a [low‐income] setting [the standard view has been that] people should not be compensated, so that they can make a voluntary decision not driven by gains that might accrue from participating in the study … [A]s much as people get worried about [payment in LMICs], it is the same as what you are seeing with people who are doing the phase one studies in Europe … [T]he students end up [serving as participants], because they want some extra money [and] because they want to be a part of something. [Scientist, Africa]

However, many interviewees recognized that CHIs involving high levels of payment in both HICs and LMICs could potentially lead to undue inducement, for example, if payment were to lead individuals to conceal important details of their medical/psychiatric history^43^ in order to avoid exclusion from participation (and thus payment). Setting appropriate levels of payment thus involves striking a balance between competing considerations. While there was not universal agreement regarding how payment levels for (LMIC) CHIs should be set, there was widespread agreement in our qualitative data that participants should, at least, be compensated for the burdens of participation (and/or for any long‐term harms that occur, although such harms are thought to be very rare). Many stakeholders suggested that decisions regarding level of payment should be informed by local community consultation and/or data from CHIs‐focused social science.^44^ It was also recognized that payment might have other as yet poorly characterized effects on, for example, (a) research institutions and competing research priorities (i.e., high levels of payment for CHIs might lead to difficulties recruiting participants for other less well‐paid studies), (b) the potential for over‐volunteering (i.e., high payment might lead some individuals to volunteer for multiple studies in such a way that it would increase risks to them and/or undermine the scientific value of a study),^45^ and/or (c) investigator‐participant interactions (e.g., researchers might treat paid participants differently than those who volunteered unpaid). Fortunately, many LMIC CHIs programs include thorough social science components, which may help to clarify the importance of such effects as well as refine decisions regarding payment and study design more generally.^46^

#### 3.2.6 Community engagement and public acceptability

A major finding of our project was that public acceptance of locally conducted CHIs is widely considered a sine qua non of ethical CHIs in LMICs. Even if a study design might be acceptable to the local ethics review committee in terms of their assessment of the balance of risks and benefits and so on, community acceptance should arguably be formally assessed. Therefore, community engagement is an essential part of setting up and maintaining CHIs capacity in LMICs because (a) CHIs represent a particularly complex and potentially controversial type of research, (b) CHIs may be particularly unfamiliar in some LMIC communities, and (c) controversy regarding CHIs may undermine local (and/or international) confidence in research and public health more generally.^47^ As one scientist summarized:

[CHIs] research is not well understood and [these studies are] introducing a very different way of doing research that has got its own potential to create rumors and greater mistrust in research. … So we just need to make sure that we are doing things the way we should. [Scientist, Africa]

Several interviewees highlighted the importance of recognizing that, ideally, engagement does not merely entail researchers informing communities about planned or on‐going research, but should be a two‐way process from which researchers could also learn about community perspectives, suggestions, concerns, etc. Such engagement activities may help to improve mutual understanding between community members, participants, investigators, and ethics review committees, as well as refine study designs and obviate controversies that could undermine important research and public health programs.

## 4 CONCLUSIONS

There are ethical and scientific reasons in favor of conducting endemic LMIC CHIs in order to address the persistently high burden of infectious diseases in disadvantaged populations. In many cases, carefully conducted endemic LMIC CHIs will lead to results that are more relevant to high‐risk target populations. Furthermore, well‐designed endemic‐region CHIs may have a more favorable profile of risks and benefits for participants (as compared with those in non‐endemic CHIs), and potentially other benefits related to improving local research capacity. However, controversies persist regarding third‐party risks, and these should be carefully assessed and minimized. Meanwhile, there was evidence of a consensus among stakeholders that payment for burdensome (LMIC) CHIs is appropriate, and recognition that blanket exclusion of less educated individuals may not, in many cases, be ethically appropriate. These findings may help to inform ongoing debates in research ethics regarding risk‐benefit analysis, payment, and consent. Finally, community consultation and social science work alongside biological scientists conducting CHIs may help to ensure the acceptability of CHIs among participants and communities and thus the ethical acceptability and sustainability of this type of research.

## CONFLICT OF INTEREST

Euzebiusz Jamrozik and Michael J Selgelid have been involved and/or played leading roles in World Health Organization Working Groups developing guidance on the ethics of human challenge studies.

## Footnotes

^1^ Roestenberg, M., Kamerling, I. M. C., & de Visser, S. J. (2018). Controlled human infections as a tool to reduce uncertainty in clinical vaccine development.*Frontiers in Medicine*, 5. p.297; Sauerwein, R. W., Roestenberg, M., & Moorthy, V. S. (2011). Experimental human challenge infections can accelerate clinical malaria vaccine development. *Nature Reviews Immunology, 11* (1), 57–64.

^2^ Roestenberg, M., Hoogerwerf, M.‐A., Ferreira, D. M., Mordmüller, B., & Yazdanbakhsh, M. (2018). Experimental infection of human volunteers. *The Lancet Infectious Diseases, 18* (10), e312–e322. http://dx.doi.org/10.1016/s1473-3099(18)30177-4

^3^ Gutmann, A., & Wagner, J. (2012). Ethically impossible: STD research in Guatemala from 1946 to 1948. Presidential Commission for the Study of Bioethical Issues. https://bioethicsarchive.georgetown.edu/pcsbi/node/654.html; Tsuchiya, T. (2008). The imperial Japanese experiments in China. In J. E. Ezekiel, C. G. Christine, A. C. Robert, K. L. Reidar, G. M. Franklin, & David, D. W (Eds.), Oxford, UK: Published by Oxford University Press *The Oxford textbook of clinical research ethics* (pp. 31–45); Weindling, P. J. (2008). The Nazi medical experiments. In J. E. Ezekiel, C. G. Christine, A. C. Robert, K. L. Reidar, G. M. Franklin, & David, D. W (Eds.), Oxford, UK: Published by Oxford University Press *The Oxford textbook of clinical research ethics* (pp. 18–30).

^4^ Jamrozik, E., & Selgelid, M. J. (2020). *Human challenge studies in endemic settings: Ethical and regulatory issues.* Cham, Switzerland: SpringerBriefs(Ethics).

^5^ Bambery, B., Selgelid, M., Weijer, C., Savulescu, J., & Pollard, A. J. (2015). Ethical criteria for human challenge studies in infectious diseases. *Public Health Ethics, 9* (1), 92–103; Hope, T., & McMillan, J. (2004). Challenge studies of human volunteers: Ethical issues. *Journal of Medical Ethics, 30*(1), 110–116.

^6^ Evers, D. L., Fowler, C. B., Mason, J. T., & Mimnall, R. K. (2015). Deliberate microbial infection research reveals limitations to current safety protections of healthy human subjects. *Science and Engineering Ethics, 21* (4), 1049–1064.

^7^ Jamrozik & Selgelid, op. cit. note 4.

^8^ Baay, M. F.D., Richie, T. L., Neels, P., Cavaleri, M., Chilengi. R., Diemert. D., … Adrian, W. (2019). Human challenge trials in vaccine development, Rockville, MD, USA, September 28–30, 2017. *Biologicals, 61*, 85–94. http://dx.doi.org/10.1016/j.biologicals.2018.02.002

^9^ Gordon, S. B., Rylance, J., Luck, A., Jambo, K., Ferreira, D. M., Manda‐Taylor, L., … Mlombe, Y. (2017). A framework for Controlled Human Infection Model (CHIM) studies in Malawi: Report of a Wellcome Trust workshop on CHIM in Low Income Countries held in Blantyre, Malawi. *Wellcome Open Research, 2,*70. http://dx.doi.org/10.12688/wellcomeopenres.12256.1

^10^ Nevertheless, there might be some cases in which HIC CHIs are adequately generalizable to LMIC populations at risk. Recent typhoid vaccine research, for example, has shown that challenge study results in non‐immune adult UK CHI participants were highly generalizable to field trial results in children in endemic LMICs; see Shakya, M., Colin‐Jones, R., Theiss‐Nyland, K., Voysey, M., Pant, D., Smith, N., … TyVAC Nepal Study Team (2019). *New England Journal of Medicine, 381*(23), 2209–2218

^11^ Baay et al., op. cit. note 8; Gordon et al., op. cit. note 9; Gibani, M. M., Jin, C., Darton, T. C., & Pollard, A. J. (2015). Control of invasive Salmonella disease in Africa: Is there a role for human challenge models? *Clinical Infectious Diseases, 61*(Suppl. 4), S266–S271.

^12^ Jamrozik & Selgelid, op. cit. note 4.

^13^ Ibid.

^14^ We are grateful to participants at two meeting in June 2019: (a) Workshop: An ethical framework for human challenge studies (organized by A/Prof. Seema Shah) and (b) Guidance Development Meeting regarding the ethics of human challenge studies (convened by the WHO Global Health Ethics Unit).

^15^ Bambery et al., op. cit. note 5.

^16^ Selgelid, M., & Jamrozik, E. (2018). Ethical challenges posed by human infection challenge studies in endemic settings. *Indian Journal of Medical Ethics*, *III*, (4), 274–278. http://dx.doi.org/10.20529/ijme.2018.073

^17^ Herrington, D. A., Van De Verg, L., Formal, S. B., Hale, T. L., Tall, B. D., Cryz, S. J., … Levine, M. M. (1990). Studies in volunteers to evaluate candidate Shigella vaccines: further experience with a bivalent Salmonella typhi‐Shigella sonnei vaccine and protection conferred by previous Shigella sonnei disease. *Vaccine*, *8*(4), 353–357. http://dx.doi.org/10.1016/0264-410x(90)90094-3

^18^ Shah, S. K., Kimmelman, J., Lyerly, A. D., Lynch, H. F., McCutchan, F., Miller, F. G., … Zorilla, C. (2017). Ethical considerations for Zika virus human challenge trials. https://www.niaid.nih.gov/sites/default/files/EthicsZikaHumanChallengeStudiesReport2017.pdf

^19^ Selgelid, M. (2013). The ethics of human microbial challenge (conference paper). Paper presented at the Controlled human infection studies in the development of vaccines and therapeutics conference, Jesus College, Cambridge, UK.

^20^ Hodgson, S. H., Juma, E., Salim, A., Magiri, C., Njenga, D., Molyneux, S., … Marsh, K. (2015). Lessons learnt from the first controlled human malaria infection study conducted in Nairobi, Kenya. *Malaria Journal*, *14*(1). http://dx.doi.org/10.1186/s12936-015-0671-x

^21^ LMIC CHIs usually screen participants using similar criteria to HIC CHIs, resulting in the exclusion of those with significant comorbidities; the degree to which such individuals can subsequently access care for conditions diagnosed by such screening and the duties investigators might have to assist individuals diagnosed in this manner raise important ethical issues common to (LMIC) research more generally.

^22^ Pitisuttithum, P., Cohen, M. B., Phonrat, B., Suthisarnsuntorn, U., Bussaratid, V., Desakorn, V., … Schiff, G. M. (2002). A human volunteer challenge model using frozen bacteria of the new epidemic serotype, V. cholerae O139 in Thai volunteers. *Vaccine, 20*(5–6), 920–925.

^23^ Lell, B., Mordmüller, B., Dejon, A. J.‐C., Honkpehedji, J., Zinsou, J., Mengue, J. B., … Kremsner, P. G. (2018). Impact of Sickle Cell Trait and Naturally Acquired Immunity on Uncomplicated Malaria after Controlled Human Malaria Infection in Adults in Gabon. *The American Journal of Tropical Medicine and Hygiene*, *98*(2), 508–515. http://dx.doi.org/10.4269/ajtmh.17-0343

^24^ It may also be ethically important to purposefully recruit individuals with such traits for CHIs that involve testing interventions that (if licensed) would be intended for use in such (sub‐)populations, since the safety and efficacy of a given intervention may be different in certain groups.

^25^ Importantly, it should not be assumed that anyone living in a country in which a pathogen is being actively transmitted (in part of the country) is at risk of infection on a day‐to‐day basis (note, for example, that at least one falciparum malaria CHIs in Kenya and several vivax malaria CHIs in Colombia actually took place in cities in which malaria is not endemic; see Hodgson et al. op. cit. note 20 and Herrera et al. op. cit. note 33).

^26^ With the exception of pathogens such as dengue, for which the sequence of infections with different strains influences the probability of severe disease (see Selgelid & Jamrozik op. cit. note 16).

^27^ Lederer, S. E. (2008). Walter Reed and the yellow fever experiments. In J. E. Ezekiel, C. G. Christine , A. C. Robert , K. L. Reidar, G. M. Franklin, & D. W. David (Eds.), *The Oxford textbook of clinical research ethics* (pp. 9–17).

^28^ A retrospective serology study of the Willowbrook population suggested that the background risk of hepatitis A was around 90% (i.e., a high background risk, meaning that increased risk of infection with this virus during the studies would arguably have been relatively low); see Krugman, S. (1986). The Willowbrook hepatitis studies revisited: Ethical aspects. *Reviews of Infectious Diseases, 8*(1), 157–162. For critical analysis of the case see Robinson, W. M., & Unruh, B. T. (2008). The hepatitis experiments at the Willowbrook State School. In *The Oxford textbook of clinical research ethics*, (pp. 80–85).

^29^ Rothman, D. J. (1982). Were Tuskegee & Willowbrook 'Studies in Nature'?. *The Hastings Center Report, 12*(2), 5. http://dx.doi.org/10.2307/3561798

^30^ Jamrozik, E., & Selgelid, M. J. (2016). Ethics, climate change and infectious disease. In C. C. Macpherson (Ed.), *Bioethical insights into values and policy* (pp. 59–75). Cham, Switzerland: Springer; Commission on Health Research for, D. (1990). *Health research: Essential link to equity in development*. New York, NY: Oxford University Press.

^31^ Darton, T. C., Blohmke, C. J., Moorthy, V. S., Altmann, D. M., Hayden, F. G., Clutterbuck, E. A., … Pollard, A. J. (2015). Design, recruitment, and microbiological considerations in human challenge studies. *The Lancet Infectious Diseases, 15*(7), 840–851.

^32^ Battin, M. P., Francis, L. P., Jacobson, J. A., & Smith, C. B. (2008). The ethics of research in infectious disease: Experimenting on this patient, risking harm to that one. In P. B. Margaret , P. F. Leslie , A. J. Jay , and B. S. Charles (Eds.), *The patient as victim and vector: Ethics and infectious disease*. Oxford, UK: Oxford University Press.

^33^ Hodgson et al., op. cit. note 20; Herrera, S., Fernández, O., Manzano, M. R., Murrain, B., Vergara, J., Blanco, P., … Chen‐Mok, M. (2009). Successful sporozoite challenge model in human volunteers with Plasmodium vivax strain derived from human donors. *The American Journal of Tropical Medicine and Hygiene, 81*(5), 740–746.

^34^ Pitisuttithum, P. (2018). Controlled human infection model (Workshop presentation). Paper presented at the Towards a new ethical framework for the use of human challenge studies on emerging infectious diseases conference, Brocher Foundation.

^35^ Jamrozik, E., & Selgelid, op. cit. note 4.

^36^ Hodgson, S. H., Juma, E., Salim, A., Magiri, C., Kimani, D., Njenga, D., … Awuondo, K. (2014). Evaluating controlled human malaria infection in Kenyan adults with varying degrees of prior exposure to Plasmodium falciparum using sporozoites administered by intramuscular injection. *Frontiers in Microbiology, 5*, 686; Shekalaghe, S., Rutaihwa, M., Billingsley, P. F., Chemba, M., Daubenberger, C. A., James, E. R., … Huber, E. (2014). Controlled human malaria infection of Tanzanians by intradermal injection of aseptic, purified, cryopreserved Plasmodium falciparum sporozoites. *The American Journal of Tropical Medicine and Hygiene, 91*(3), 471–480.

^37^ Herrera et al., op. cit. note 33; for example, in the Colombian malaria CHIs cited here, one participant who also worked as a paramedic was strongly suspected to have self‐treated with antimalarials after challenge, thus undermining the scientific value of their participation in the experiment. In another case that attracted media attention, a nurse participating in a UK malaria challenge study absconded from the study to travel to another country without informing the research team.

^38^ Shekalaghe et. al. op. cit. note 36; Jongo, S. A., Shekalaghe, S. A., Church, L. W. P., Ruben, A. J., Schindler, T., Zenklusen, I., … Mkindi, C. (2018). Safety, immunogenicity, and protective efficacy against controlled human malaria infection of Plasmodium falciparum sporozoite vaccine in Tanzanian adults. *The American Journal of Tropical Medicine and Hygiene, 99*(2), 338–349.

^39^ Hodgson et al. op. cit. note 20.

^40^ Njue, M., Njuguna, P., Kapulu, M. C., Sanga, G., Bejon, P., Marsh, V., … Kamuya, D. (2018). Ethical considerations in controlled human malaria infection studies in low resource settings: Experiences and perceptions of study participants in a malaria challenge study in Kenya. *Wellcome Open Research*, *3*; London, A. J. (2005). Undue inducements and reasonable risks: Will the dismal science lead to dismal research ethics? *The American Journal of Bioethics, 5*(5), 29–32. https://doi.org/10.12688/wellcomeopenres.14439.2

^41^ Hodgson et al. (2014), op. cit. note 36.; Hodgson et al. (2015), op. cit. note 20.

^42^ Njue et al., op. cit. note 40.

^43^ E.g., in cases where such details would be associated with increased risks and/or undermine the scientific benefit of participation. At least one UK/European researcher was aware of an unpublished case where a participant’s undisclosed psychiatric history was thought to have contributed to a significant adverse event during a CHI.

^44^ Njue et al., op. cit. note 40; Njue, M., Kombe, F., Mwalukore, S., Molyneux, S., & Marsh, V. (2014). What are fair study benefits in international health research? Consulting community members in Kenya. *PLoS ONE*, *9*(12).

^45^ Allen, C., Francis, G., Martin, J., & Boyce, M. (2017). Regulatory experience of TOPS: An internet‐based system to prevent healthy subjects from over‐volunteering for UK clinical trials. *European Journal of Clinical Pharmacology, 73* (12), 1551–1555.

^46^ Njue et al., op. cit. note 40.

^47^ El Setouhy, M., Agbenyega, T., Anto, F., Clerk, C. A., Koram, K. A., English, M., … Kumwenda, N. (2004). Moral standards for research in developing countries from" reasonable availability" to" fair benefits". *The Hastings Center Report, 34*(3), 17–27; Kenya Medical Research Institute (KEMRI). (2018). Response to an article carried in The Standard [Press release]. https://www.kemri.org/wp-content/uploads/2019/11/RESPONSE-TO-AN-ARTICLE-CARRIED-IN-THE-STANDARD-ON-MALARIA-TRIALS.pdf

